# Characteristics of Patients Showing Discrepancy Between Bruch’s Membrane Opening-Minimum Rim Width and Peripapillary Retinal Nerve Fiber Layer Thickness

**DOI:** 10.3390/jcm8091362

**Published:** 2019-09-01

**Authors:** Hyun-kyung Cho, Changwon Kee

**Affiliations:** 1 Department of Ophthalmology, Gyeongsang National University Changwon Hospital, School of Medicine, Gyeongsang National University, Changwon 51472, Korea; 2 lnstitute of Health Sciences, School of Medicine, Gyeongsang National University, Jinju 52727, Korea; 3 Department of Ophthalmology, Samsung Medical Center, School of Medicine, Sungkyunkwan University, Seoul 06351, Korea

**Keywords:** Bruch’s membrane opening minimum rim width, diagnosis of glaucoma, glaucoma, optical coherence tomography, retinal nerve fiber layer

## Abstract

*Background:* To investigate clinical characteristics of patients showing discrepancy between Bruch’s membrane opening minimum rim width (BMO-MRW) and peripapillary retinal nerve fiber layer (RNFL) thickness. Correlation with the visual field (VF) was also inspected. *Methods:* In this prospective, cross-sectional study, 106 eyes (106 subjects) showing normal BMO-MRW classification but abnormal RNFL classification were included. All patients underwent confocal scanning laser ophthalmoscopy, spectral-domain optical coherence tomography, and standard automated perimetry. *Results:* Clinical characteristics were as follows: mean age: 52.79 ± 14.75 years; spherical equivalent (SE), −2.52 ± 3.48 diopter (D); SE < −5.0 D, 34 (32.1%) eyes; large disc (>2.43 mm^2^), 40.6%; small disc (<1.63 mm^2^), 12.5%; VF index, 96.72 ± 9.58%; mean deviation, −1.74 ± 3.61 dB; β-peripapillary atrophy (PPA), 96.2%; γ-PPA, 75.5%. Majority (86.1%) of these cases demonstrated normal (71.3%) or borderline (14.9%) on VF. Temporal and nasal RNFL showed significant differences among disc size subgroups (all *p* < 0.05). Nasal RNFL was significantly thicker in a large disc group than other subgroups. Temporal, superotemporal, inferotemporal, inferonasal RNFL, and superior RNFL peak location showed significant differences (all *p* < 0.05) among SE subgroups. Temporal RNFL was significantly thicker in the high myopia group than other subgroups. *Conclusions:* Temporalization of RNFL peaks in myopia and nasalization of RNFL peaks in large disc that display abnormal classifications might show normal classification of BMO-MRW. These findings of discrepancy between classifications should be considered in the diagnosis of early glaucoma.

## 1. Introduction

**Figure 1:**
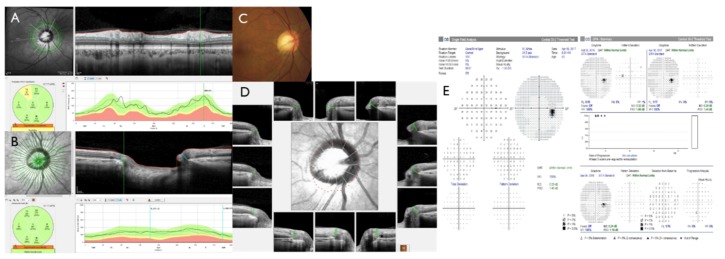
Representative case of a large optic disc. A 54-year-old male with disc area of 2.83 mm^2^ (normal range: 1.63~2.43) and spherical equivalent of -4.88 Diopter showed abnormal classification at the superotemporal (ST) sector (**A**). However, Bruch’s membrane opening minimum rim width (BMO-MRW) showed normal classification at global and at six sectors (**B**). Fundus photography showed no focal neuroretinal rim thinning or notch with β-peripapillary atrophy (PPA) in a large disc (**C**). The BMO overview showed round BMO-based optic disc margin (dotted red line) including the PPA area (**D**). Note that the superior curve and peak of peripapillary retinal nerve fiber layer (RNFL) shifted to a more nasal position than normative data (A). This yielded abnormal classification of the ST sector of RNFL or, in this case, of a large disc. Glaucoma hemifield test of the visual field was shown within normal limits in four consecutive tests (**E**).

**Figure 2:**
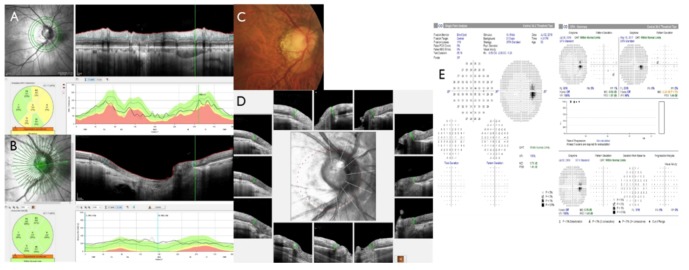
Representative case of myopia. A 53-year-old male with a spherical equivalent of ×7.75 Diopter and disc area of 2.58 mm^2^ (normal range: 1.63~2.43) showed an abnormal classification at inferotemporal (IT) and superonasal (SN) sectors with a global classification of borderline (**A**). However, Bruch’s membrane opening minimum rim width (BMO-MRW) showed normal classification at global areas and at six sectors (**B**). Fundus photography showed peripapillary atrophy (PPA) and tilted myopic optic disc (**C**). Spectral-domain optical coherence tomography showed β-PPA along with γ-PPA as border tissue without Bruch’s membrane (**B**,**D**). The BMO overview showed round BMO-based optic disc margin (dotted red line) including the PPA area. Note that the superior curve and peak of peripapillary retinal nerve fiber layer (RNFL) shifted to a more temporal position than normative data (A). This yielded abnormal classification of the SN sector of RNFL for myopia. Glaucoma hemifield the test of the visual field, which showed within normal limits in four consecutive tests in this case (**E**).

Glaucoma involves the damage of retinal ganglion cells (RGC), which causes thinning of the neuro-retinal rim and the retinal nerve fiber layer (RNFL) that leads to visual field (VF) loss [1]. Assessment of structural change is more significant than assessing functional defects in the diagnosis of early glaucoma [2,3] since structural defects may precede VF deficit of function at a detectable level [4,5,6]. Since structural change is minimal in glaucoma suspect or early stage of glaucoma, results from different structural examination may not be consistent and discrepant with each other. For instance, parameters from optical coherence tomography (OCT) such as Bruch’s membrane opening minimum rim width (BMO-MRW) and peripapillary RNFL thickness may demonstrate discrepancy in the same patient. If one parameter of the structural test indicates normalcy while another indicates an abnormal factor, this discrepancy may confuse clinicians in diagnosing early glaucoma. Clinical interpretation of RNFL and BMO-MRW abnormalities often relies on the diagnostic classification report, which classifies global and sectoral measurements into three categories (within normal limits, borderline, and outside normal limits) by taking a reference from normative data installed in the OCT instrument (Figure 1A,B, Figure 2A,B). Glaucoma at an early stage is of particular importance since the decision of whether to initiate lifetime treatment is needed.

Bruch’s membrane opening minimum rim width (BMO-MRW) has been recently suggested in the assessment of the optic nerve head [7,8,9,10,11]. It measures the minimum distance from the BMO to the internal limiting membrane (ILM, Figure 1B,D). BMO-MRW provides geometrically more precise evaluation of the neuro-retinal rim than other previously existing ophthalmic inspection and also consistent optic disc borders [7,8,9,12]. Recent studies have also demonstrated that BMO-MRW had a greater diagnostic outcome than other previously existing neuro-retinal rim parameters for glaucoma [13,14,15]. It has been revealed that BMO-MRW has better correlation with the VF than RNFL thickness or other optic nerve head parameters [15,16].

The aim of this prospective, cross-sectional study was to investigate clinical characteristics of patients showing a discrepancy between Bruch’s membrane opening minimum rim width (BMO-MRW) and peripapillary retinal nerve fiber layer (RNFL) thickness. We also inspected the VF test results in these subjects. Discrepancy between RNFL and BMO-MRW or definite consensus on diagnostic criteria integrating both parameters in glaucoma has not been reported yet. Thus, we aimed to determine in which case that BMO-MRW (the new parameter) can show normal classification while RNFL (the conventional parameter) shows abnormal classification. We tried to find in which case BMO-MRW may provide a more reliable color code classification than RNFL when clinicians may misdiagnose glaucoma based on the possible false positive RNFL color code classification in early glaucoma.

## 2. Experimental Section

### 2.1. Subjects

Among 111 eyes showing normal BMO-MRW classification but abnormal RNFL classification who were evaluated by Spectralis Glaucoma Module Premium Edition (Heidelberg Engineering, Germany) in the glaucoma clinic at Gyeongsang National University Changwon Hospital, 106 eyes (106 subjects) were included. Normal classification shows green while abnormal classification shows yellow or red on Spectralis spectral-domain optical coherence tomography (Glaucoma Module Premium Edition, Heidelberg Engineering, Germany) (Figure 1A,B, Figure 2A,B). Only one eye was chosen randomly, if both eyes satisfied the criteria of inclusion. All subjects performed standard ophthalmic examinations including confocal scanning laser ophthalmoscopy (HRT3, Heidelberg Engineering, Germany), Spectralis spectral-domain OCT, and standard automated perimetry (HFA model 840, Humphrey Instruments Inc, San Leandro, CA, USA). Five subjects were excluded according to the following exclusion criteria. The criteria include poor images due to blinking or poor fixation, history of intraocular surgery other than uneventful phacoemulsification, history of acute angle closure, or other optic neuropathy other than glaucoma that may affect the thickness of RNFL or BMO-MRW (ex., acute ischemic optic neuritis, optic neuritis).

Among 106 subjects included in the final analysis, 101 subjects had a reliable VF test. A reliable VF test was defined as follows: fixation loss less than 20%, false positive rate <15%, and false negative rate <15%. Ten subjects had poor image of confocal scanning laser ophthalmoscopy (CSLO) who were excluded from CSLO analysis. A total of 96 subjects had reliable CSLO tests. However, all included subjects (*n* = 106) had reliable OCT tests.

This prospective, cross-sectional study was conducted in accordance to the tenets of the Declaration of Helsinki. The present study was approved by the Institutional Review Board of Gyeongsang National University Changwon Hospital, Gyeongsang National University School of Medicine (GNUCH 2018-05-016, approved on 4 June, 2018). Written informed consents were obtained from all patients enrolled in this study.

### 2.2. Optical Coherence Tomography

Imaging of Spectral-domain OCT was conducted using the Glaucoma Module Premium Edition. Radial B-scans of 24 were acquired for BMO-MRW. Scan circle of 3.5 mm in diameter among three scan circles (3.5, 4.1, and 4.7 mm in diameter) was used for peripapillary RNFL thickness. Well-centered scans with precise retinal segmentation and a quality score of more than 20 were adopted. Data collection and analyses were achieved with regard to an individual specific axis (Fovea-BMO axis, FoBMO axis), which is the axis between the BMO center and fovea. The FoBMO axis could result in more precise sectoral analysis regarding cyclotorsion of individuals and enables a more correct assessment with a normative database than the conventional technique [7].

### 2.3. Confocal Scanning Laser Ophthalmoscopy and Perimetry

CSLO imaging using HRT3 software (Heidelberg Engineering, Germany) was carried out by an experienced technician. Exclusion criteria were as follows: mean pixel height standard deviation >30 mm, decentration of images, under-illumination, and moving artifacts for quality of scans. Subjects were classified on the basis of disc area obtained by CSLO as a large disc (>2.43 mm^2^), regular disc (1.63–2.43 mm^2^), and small disc (<1.63 mm^2^) on the basis of a normative data range from installed software. Humphrey Field Analyzer (HFA model 840, Humphrey Instruments Inc, San Leandro, CA, USA) was used for the VF test with central 30-2 program of the Swedish Interactive Threshold Algorithm standard strategy.

### 2.4. Statistical Analysis

We compared continuous variables including RNFL thickness or RNFL peak location, disc area, spherical equivalent among the three subgroups on the basis of optic disc size or spherical equivalent using the Kruskal-Wallis test. We also compared categorical variables such as RNFL classification or glaucoma hemifield test classification among the three subgroups using the Kruskal-Wallis test. To compare the two groups, the Kruskal-Wallis test with the Donn’s post-hoc test was used. Statistical significance was considered when *p* < 0.05. Statistical analyses were conducted using SPSS software version 24.0 (IBM Corp., Chicago, IL, USA).

## 3. Results

### 3.1. Baseline Characteristics

A total of 106 eyes (106 subjects) were included in the final analysis. Mean age of subjects was 52.79 ± 14.75 years. Among these subjects, 66 (62.3%) were men and 40 (37.7%) were women. Eleven (10.4%) subjects had glaucoma family history. The mean spherical equivalent (SE) of all subjects was −2.52 ± 3.48 diopter (D). However, 26 (24.5%) subjects had SE less than −6.0 D and 34 (32.1%) had SE less than −5.0 D. Baseline intraocular pressure was 15.20 ± 3.17 mmHg and central corneal thickness (CCT) was 551.19 ± 38.26 um. β-peripapillary atrophy (PPA) and γ-PPA assessed with spectral-domain OCT were observed in 96.2% (102/106) of eyes and in 75.5% (80/106) of eyes, respectively. The mean visual field index (VFI) was 96.72 ± 9.58%. Mean deviation (MD) and pattern standard deviation (PSD) were −1.74 ± 3.61dB and 2.86 ± 1.89 dB, respectively. VF test results were within normal limits (WNL) in 71.3% (72/101), borderline (BL) in 14.9% (15/101), and outside normal limits (ONL) in 13.8% (14/101) (Table 1). VF test results of either WNL or ONL were determined with Anderson and Patella criteria including Glaucoma Hemifield Test results [17].) VF results of BL was determined with Glaucoma Hemifield Test results from the Humphrey Field Analyzer.

Optic disc parameters were obtained with CSLO (HRT3). Mean disc area was 2.35 ± 0.68 mm^2^. However, 40.6% (39/96) of eyes had a large disc (>2.43 mm^2^) and 12.5% (12/96) had a small disc (<1.63 mm^2^) while 46.9% (45/96) of eyes had a regular disc (1.63~2.43 mm^2^). This classification of optic disc size was based on the normative range of HRT3 software. Parameters of the optic nerve head are shown in detail in Table 2. Cup/disc ratios of horizontal and vertical shape were 0.62 ± 0.22 and 0.56 ± 0.20, respectively. Rim volume was 0.37 ± 0.15 mm^3^, which was within the normal range (0.30~0.61 mm^3^). 

### 3.2. Retinal Nerve Fiber Layer and Bruch’s Membrane Opening Minimum Rim Width

RNFL and BMO-MRW were measured by Spectralis SD-OCT Glaucoma Module Premium Edition. Mean global RNFL thickness was 93.07 ± 10.09 µm. Regarding color classification, 75.5%, 13.2%, and 11.3% showed WNL, BL, and ONL, respectively. RNFL thickness values of the other six Garway-Heath sectors are shown in Table 3. Average superior and inferior peak locations were at 79.29 ± 15.04° and 297.67 ± 15.98°, respectively, on the TSNIT graph.

Mean BMO area was 2.68 ± 0.74 mm^2^. The BMO area acquired by OCT and disc area obtained by HRT demonstrated a strong correlation (Spearman’s rho 0.680, *p* < 0.001). Global BMO-MRW in average was 262.04 ± 52.87 um. All eyes (100%) had color classification of WNL. BMO-MRW values of the other six Garway-Heath sectors are shown in Table 3. The mean BMO-fovea angle was −5.78 ± 3.87. The mean quality scores of RNFL and BMO-MRW were very high (30.17 ± 3.39 and 32.92 ± 2.81, respectively).

### 3.3. Retinal Nerve Fiber Layer According to Optic Disc Size

According to optic disc size based on disc area, participants were divided into three subgroups: large (subgroup 1, 2.99 ± 0.51 mm^2^), regular (subgroup 2, 2.06 ± 0.23 mm^2^), and small disc (subgroup 3, 1.39 ± 0.22 mm^2^). There was a significant difference in disc area among the three groups (Kruskal-Wallis test, *p* < 0.0001). Temporal and nasal RNFL showed significant differences among disc size groups (Kruskal-Wallis test, *p* = 0.023, *p* < 0.0001, respectively) (Table 4). Temporal RNFL was significantly thicker in subgroup 3 than that in subgroup 2 (Kruskal-Wallis test with Donn’s post-hoc test, *p* = 0.019). Nasal RNFL was significantly thinner in subgroup 3 than that in subgroup 1 or subgroup 2 (Kruskal-Wallis test with Donn’s post-hoc test, *p* < 0.0001, *p* = 0.015, respectively). There was also a significant difference in color classification for nasal RNFL among the three subgroups (Kruskal-Wallis test, *p* = 0.026). Large disc had relatively thicker nasal RNFL while small disc had relatively thinner nasal RNFL and thicker temporal RNFL. Superior and inferior RNFL peak location, GHT of VF, and SE showed no significant differences among the three-disc size subgroups (Kruskal-Wallis test, all *p* > 0.05) (Table 4). A representative case is demonstrated in Figure 1.

### 3.4. Retinal Nerve Fiber Layer According to Myopia

In accordance with SE, subjects were divided into three subgroups: no to mild myopia (SE > −2.0D, −0.03 ± 1.08D, *n* = 54, subgroup 1), moderate (SE = −2.0~ −5.0D, −3.60 ± 0.98D, *n* = 18, subgroup 2), and high myopia (SE < −5.0D, −5.90 ± 3.67D, *n* = 34, subgroup 3). There was a significant difference in SE among the three groups (Kruskal-Wallis test, *p* < 0.0001). Global, temporal, superotemporal, inferotemporal, and inferonasal RNFL showed significant differences among SE groups (Kruskal-Wallis test, all *p* < 0.05) (Table 5). Global and temporal RNFLs were significantly thicker in subgroup 3 than those in subgroup 2 (Kruskal-Wallis test with Donn’s post-hoc test, *p* = 0.029 and *p* = 0.017, respectively). Temporal, superotemporal, and inferotemporal RNFLs were significantly thicker in subgroup 3 than those in subgroup 1 (Kruskal-Wallis test with Donn’s post-hoc test, all *p* < 0.05). Inferonasal RNFL was significantly thicker in subgroup 1 than in subgroup 2 (Kruskal-Wallis test with Donn’s post-hoc test, *p* = 0.017). High myopia subgroup had a relatively thicker temporal region (T, TS, TI) RNFL thickness than other subgroups. There was a significant difference in the superior RNFL peak location among the three subgroups (Kruskal-Wallis test, *p* = 0.033). The high myopia subgroup had a more temporally located superior RNFL peak than other subgroups. However, inferior RNFL peak location, disc area, and GHT of VF showed no significant differences among the three SE subgroups (Kruskal-Wallis test, all *p* > 0.005) (Table 5). A representative case is demonstrated in Figure 2.

### 3.5. Retinal Nerve Fiber Layer According to Visual Field Test Results

When RNFL was analyzed according to VF test results of WNL (*n* = 72), BL (*n* = 14), and ONL (*n* = 15), there were no significant differences among three VF subgroups in global areas and six sectors (Kruskal-Wallis test, all *p* > 0.005). RNFL classification also demonstrated no significant differences among the three VF subgroups (Kruskal-Wallis test, all *p* > 0.005). Superior and inferior peal location, disc area, and SE also revealed no significant differences among the three VF subgroups (Kruskal-Wallis test, all *p* > 0.005) (Table 6).

## 4. Discussion

To our knowledge, the current study is the first study to investigate clinical characteristics of patients showing discrepancy between BMO-MRW and peripapillary RNFL thickness. We included subjects showing normal color code classification of BMO-MRW and abnormal color code classification of RNFL thickness and inspected the VF test results in these subjects. We aimed to see in which case RNFL might show a false positive finding, but BMO-MRW would not show this in early glaucoma. Furthermore, we aimed to see in which case BMO-MRW may provide more reliable classification than RNFL and apply these findings in clinical diagnosis of early glaucoma. Since there is a lack of any consensus regarding how OCT diagnostic classification should be interpreted by integrating RNFL and BMO-MRW, our study provides a perspective on the diagnosis of early glaucoma combining both parameters. We found that nasalization of RNFL peaks and, subsequently, thicker nasal RNFL in a large disc might display abnormal classifications based on RNFL, even though they might show normal classification based on BMO-MRW. We also found that temporalization of RNFL peaks and, accordingly, thicker temporal RNFL in myopia might display abnormal classifications based on RNFL. However, they might show normal classification based on BMO-MRW. Since 86.1% of these cases demonstrated normal findings on VF tests, it would be more reasonable to interpret that these cases are more likely a false positive color code classification of RNFL than false negative color code classification of BMO-MRW.

These two cases are important for clinicians because they represent challenging cases in diagnosing early glaucoma. In large discs, the rim of neuroretina on fundus photography seems thinned and the cup of the optic nerve head looks large while the number of RGC axons is equivalent to or greater than regularly-sized discs. Thus, the eyes of large discs demonstrate physiologically large cupping of the optic disc that might lead to a suspicion of glaucoma and possibly unnecessary treatment decisions [18,19,20,21]. Myopia also presents distinctive challenges in diagnosing glaucoma. Greater prevalence of glaucoma in myopia has been reported in population-based studies [22,23,24]. Myopia has been reported as a risk factor for open angle glaucoma more frequently in Asians than in other races, according to population-based studies [24]. Myopic eyes show shallow cupping along with sometimes a pale neuro-retinal rim, which makes the assessment of the optic disc difficult. Structural tests can demonstrate abnormal results in myopic eyes since normative databases consist of subjects with a relatively low refractive error [25,26]. RNFL thickness and cup-to-disc ratio measured by commercial OCT and CSLO have been revealed to be less useful to differentiate glaucoma and non-glaucoma individuals with high myopia than subjects from normal population [27]. A number of studies have revealed that RNFL thinning was associated with myopia [28,29,30].

The current study did not perform area under the receiver operating characteristic (ROC) curve (AUC) analysis because AUCs were typically compared across a wide range of false-positive values (i.e., between 0% and 100%). Sensitivities derived from the ROC curve are often compared at a specificity of 90% or 95% [13,31,32]. Sensitivities of global BMO-MRW and global RNFL thickness determined from diagnostic classification analysis were compared at higher specificities (between 98.7% and 100%) [33]. High specificities were observed in diagnostic classification analysis because the first percentile and the fifth percentile of normative BMO-MRW/RNFL thickness data were used to define BMO-MRW/RNFL thickness abnormalities [33].

BMO is the external border of the neural tissue at the optic nerve head and RGC axons pass through BMO [12]. BMO-MRW provides geometrically more precise evaluation of the neuro-retinal rim than other existing ophthalmic analysis [7,8,9,12]. BMO-MRW has been reported to have an advantage to accurately indicate the amount of neuro-retinal rim tissue from the optic disc [34].

Previous studies have revealed that the structure-function relationship is better with the BMO-MRW parameter than other conventional OCT-based and CSLO-based parameters [15,35,36]. Pollet-Villard et al. [15] have demonstrated that the structure-function relationship is significantly greater with BMO-MRW than other optic disc OCT parameters including neuro-retinal rim thickness. It is also stronger than RNFL thickness. Enders et al. [35] have concluded that BMO-MRW seems to indicate the structure-function relationship better than RNFL thickness and rim area in CSLO. Recently, the structure-function relationship in lamina cribrosa (LC)-derived parameters has been inspected. Lopes et al. [36] have found that BMO-MRW has better structure-function correlations than LC-derived parameters including prelaminar neural tissue thickness and area, anterior LC depth, and LC thickness and area. These findings partly support our study results that most (86.1%) included subjects showing normal classification of BMO-MRW and abnormal classification of RNFL demonstrated normal findings on VF tests.

The appearance of neuro-retinal rim in large discs with large cupping is different from that of regular-sized optic discs. Cupping is more vertical at the neuro-retinal rim wall, and a punched-out shape mimicking neuro-retinal rim thinning of glaucoma [37,38]. It has been demonstrated that the distribution of RNFL in subjects with large disc was altered when compared with normal controls [38]. Lee et al. [38] found that peaks of the RNFL graph at a superior and inferior location shifted to a more nasal location and thicker nasal RNFL in subjects with a large disc than the control. Results of our study also demonstrated findings concordant with the study by Lee et al. [38]. Subjects with large disc subgroup showed significantly thicker nasal RNFL than other subgroups. Classification of RNFL also showed a significant difference in the nasal sector among the three subgroups based on disc size in our study. Therefore, RNFL classification of OCT-based on a normative database of built-in software can lead to clinicians, especially non-glaucoma specialists, to misinterpret this false-positive finding in a large disc [38]. It can also lead to unnecessary treatment that can yield needless cost and efforts.

It has been recently demonstrated that BMO-MRW was a useful parameter in diagnosing glaucoma in large discs [35]. Enders et al. [35] found that global BMO-MRW correlated better with VF test results (Spearman’s Rho (ρ) = 0.71, *p* < 0.001) than with RNFL (ρ = 0.52, *p* < 0.001) or CSLO rim area (ρ = 0.63, *p* < 0.001) in large discs. They revealed that BMO-MRW had greater diagnostic performance to differentiate glaucoma patients from normal subjects in macro-discs than RNFL thickness or rim area in CSLO [34]. The study by Enders et al. [35] also supports our results about the subgroup of a large disc.

Myopia also influences RNFL distribution. Therefore, patients with myopia can show abnormal results on structural tests based on normative data in OCT from individuals with a low refractive error [25,26]. It has been reported that conventional RNFL analysis does not apply to eyes such as PPA, disc tilt, and high myopia [39,40,41]. Hwang et al. [42] have demonstrated that myopic eyes with optic disc tilt at the temporal region and rotation of counterclockwise direction had thicker RNFL at the temporal sector and a more temporally located superior peak position. The other study by Kang et al. [29] has demonstrated that temporal peripapillary RNFL thickness increases when axial length is increased and the spherical equivalent decreased. Results of the present study are concordant with these previously investigated studies. In the current study, the high myopia subgroup showed significantly thicker temporal RNFL than other subgroups. Superotemporal and inferotemporal RNFL were also significantly thicker in the high myopia subgroup than those in the mild myopia subgroup. Superior peak location of RNFL was also significantly different among the three subgroups, according to SE in the present study. Since myopia has an impact on RNFL distribution, classification of RNFL from built-in OCT software can show abnormal results. However, as shown in our study, BMO-MRW can demonstrate normal classification in myopia when conventional RNFL cannot reflect a true optic nerve head status (either normal or abnormal). BMO-based neuro-retinal rim analysis may be more useful for discriminating glaucoma from normal in a myopic condition, which frequently accompanies optic disc tilt and PPA than RNFL thickness. In our study, β-PPA was observed in 96.2% (102/106) of subjects and γ-PPA was found in 75.5% (80/106) of subjects. PPA also seems to affect the discrepancy of RNFL and BMO-MRW classification regardless of myopia. Even when RNFL shows abnormal classification due to PPA, BMO-MRW can display normal classification. Thus, taking this finding into consideration when assessing patients with PPA is advisable.

Rebolleda et al. have investigated and compared a false-positive color code classification of BMO-MRW and RNFL in healthy eyes with a tilted optic disc [43]. They found that the overall false-positive rate was significantly lower with the BMO-MRW map compared to both the RNFL map by Spectralis OCT and ganglion cell analysis map by Cirrus OCT [43]. Moreover, BMO-MRW provided significantly higher specificity than RNFL in tilted disc irrespective of the refractive error. It was more specific than ganglion cell analysis in subjects with a tilted disc showing moderate myopia (−2.5 to −6 D) [43]. Another study [44] has evaluated whether the new BMO-based rim analysis shows advantages over RNFL thickness in subjects with moderate myopia. It found that rim analysis of BMO-MRW showed a lower rate of false-positives in comparison with RNFL thickness in healthy moderate myopia (−3 to −6 D) [44]. These previous studies support findings of our study regarding a discrepancy between BMO-MRW and RNFL classification in myopic subgroup eyes. It has been reported that a subgroup of myopic subjects (>4 D) with glaucoma demonstrate greater correlations between MD and BMO-MRW than between MD and RNFL thickness [45]. This could partly explain why the majority of our subjects including moderate (SE −2 to −5 D) and high myopia (<−5 D) showed normal findings on VF tests.

VF test results showed ONL in 13.8% (14/101) of eyes in the present study. The RNFL defect was noticed in fundus photography with a red-free filter. It was also found on OCT with a corresponding VF defect in these cases. However, neuro-retinal rim demonstrated no focal thinning or notch. It seemed concentric. We are not sure whether these cases are true glaucoma or a secondary RNFL defect due to other unknown/undetected retinal and/or optic nerve disorder. On the contrary, a possibility of pre-perimetric glaucoma remains in those subjects showing normal VF test results. Further longitudinal studies are needed to define these conditions. However, BMO-MRW may reflect neuro-retinal rim tissue more accurately than RNFL in a glaucoma suspicion or glaucoma in an early stage.

The strength of our study is that it is the first study to provide possible factors that could influence the discrepancy between BMO-MRW and RNFL classification in the diagnosis of early glaucoma. It presents possible clinical cases that can be displayed as a false positive finding in RNFL analysis of conventional built-in software in the OCT device. It also provides useful cases of BMO-MRW (the new parameter) when it can demonstrate normal classification and WNL of the VF test. Such information is valuable to clinicians since, currently, there is no definite consensus on the diagnostic criteria between these two parameters (BMO-MRW and RNFL). The current study is also unique in that all parameters are investigated in the same ethnic population of East Asians (Korean) where myopia is relatively predominant. Myopic prevalence is greater in Asian studies [24,46,47,48] than those in white and Hispanic populations [49,50,51]. Myopia frequently causes difficulties in diagnosing glaucoma due to abnormal results on conventional structural tests.

This study also has some limitations. First, it had a hospital-based design. This study was conducted at a referral university hospital of the province. It was not a population-based study. Although this study was prospective, subjects included in the study may not represent a normal population. Another limitation was that we only included subjects who could have performed a reliable visual field test with considerable quality of OCT and CSLO images. We do not know the effect of such a selection of subjects on our results. The sample size of the present study should also be considered. The the small optic disc subgroup and the moderate myopia subgroup had a relatively small number of subjects. The total number of subjects included was 106. However, when subjects were divided into subgroups, the distribution was not even. This is due to the subjects not being recruited according to specific subgroup criteria. Instead, primary inclusion criteria were given. After that, heterogeneous subjects were divided into subgroups for analysis. This may have affected the accuracy of estimated proportions. We did not evaluate those eyes showing normal RNFL classification and abnormal BMO-MRW classification, which is the opposite case of our study inclusion. These cases may also need to be investigated to conclude our findings. In addition, the present study did not provide long-term observational data on these selected subjects. A long-term study with a large number of subjects from a multicenter is needed to observe progression changes of these subjects.

In conclusion, we found that large disc and myopia might show abnormal classifications in RNFL. However, they might show normal classification based on BMO-MRW. This may confuse clinicians in diagnosing early glaucoma. Nasalization of RNFL peaks in large disc and temporalization of RNFL peaks in myopia might show such findings. VF tests showed mainly normal findings in these cases. BMO-MRW may provide more reliable color code classification than RNFL in these cases when clinicians may misdiagnose glaucoma based on the possible false positive RNFL color code classification. Accordingly, when evaluating BMO-MRW in association with RNFL thickness, these cases might need to be taken into clinical consideration. A large number of the population-based study is required to draw definitive conclusions.

## Figures and Tables

**Table 1:** Baseline characteristics of included subjects.

Characteristics	Values
Number of subjects	106 eyes (106 subjects)
Mean Age (year)	52.79 ± 14.75
Female gender (%)	40 (37.7%)
Family history of glaucoma (%)	11 (10.4%)
Spherical equivalent (D)	−2.52 ± 3.48
<−6.0D	26 eyes (24.5%)
<−5.0D	34 eyes (32.1%)
CCT (um)	551.19 ± 38.26
Baseline IOP (mmHg)	15.20 ± 3.17
β-PPA (%)	102 eyes (96.2%)
γ-PPA (%)	80 eyes (75.5%)
VFI (%)	96.72 ± 9.58
MD (dB)	−1.74 ± 3.61
PSD (dB)	2.86 ± 1.89
GHT	WNL: 72/101 eyes (71.3%)
	BL: 15/101 eyes (14.9%)
	ONL: 14/101 eyes (13.8%)

CCT, central corneal thickness. D, diopters. IOP, intraocular pressure. VFI, visual field index. MD, mean deviation. PSD, pattern standard deviation. GHT, glaucoma hemifield test. WNL, within normal limits. BL, borderline. ONL, outside normal limits.

**Table 2:** Optic disc parameters of the subjects.

Characteristics	Values
Disc area (mm^2^)	2.35 ± 0.68 (1.63–2.43)
Large disc (>2.43 mm^2^)	2.99 ± 0.51 (39/96 eyes, 40.6%)
Regular disc (1.63~2.43 mm^2^)	2.06 ± 0.23 (45/96 eyes, 46.9%)
Small disc (<1.63 mm^2^)	1.39 ± 0.22 (12/96 eyes, 12.5%)
Cup area (mm^2^)	1.00 ± 0.65 (0.11–0.68)
Rim area (mm^2^)	1.35 ± 0.32 (1.31–1.96)
Cup/disc area ratio	0.39 ± 0.18 (0.07–0.30)
Rim/disc area ratio	0.61 ± 0.18 (0.70–0.93)
Cup volume (mm^3^)	0.28 ± 0.27 (0.01–0.18)
Rim volume (mm^3^)	0.37 ± 0.15 (0.30–0.61)
Mean cup depth	0.31 ± 0.11 (0.10–0.27)
Horizontal cup/disc ratio	0.62 ± 0.22
Vertical cup/disc ratio	0.56 ± 0.20

Measured by confocal scanning laser ophthalmoscope: 10 images excluded due to poor image quality. (Normal range).

**Table 3:** Retinal nerve fiber layer and Bruch membrane opening minimum rim width.

Characteristics	RNFL Thickness	RNFL Classification			Superior Peak Location	Inferior Peak Location
		WNL	BL	ONL		
RNFL G (µm)	93.07 ± 10.09	80 (75.5%)	14 (13.2%)	12 (11.3%)	79.29 ± 15.04	297.67 ± 15.98
RNFL T	80.01 ± 17.38	102 (96.3%)	3 (2.8%)	1 (0.9%)	(75–85)	(295–300)
RNFL TS	124.13 ± 27.05	75 (70.7%)	18 (17.0%)	13 (12.3%)		
RNFL TI	132.91 ± 24.24	46 (43.4%)	34 (32.1%)	26 (24.5%)		
RNFL N	69.76 ± 16.42	74 (69.8%)	11 (10.4%)	21 (19.8%)		
RNFL NS	112.26 ± 28.25	89 (84.0%)	14 (13.2%)	3 (2.8%)		
RNFL NI	96.89 ± 27.22	75 (70.7%)	19 (18.0%)	12 (11.3%)		
Quality score	30.17 ± 3.39					
	**BMO-MRW**	**BMO-MRW Classification**				
BMO area (mm^2^)	2.68 ± 0.74					
BMO-MRW G (um)	262.04 ± 52.87	106 (100%)				
BMO-MRW T	200.81 ± 45.01	106 (100%)				
BMO-MRW TS	257.54 ± 62.45	106 (100%)				
BMO-MRW TI	291.85 ± 61.89	106 (100%)				
BMO-MRW N	274.54 ± 65.69	106 (100%)				
BMO-MRW NS	290.48 ± 62.19	106 (100%)				
BMO-MRW NI	311.69 ± 65.62	106 (100%)				
BMO-fovea angle°	−5.78 ± 3.87					
Quality score	32.92 ± 2.81					

RNFL, retinal nerve fiber layer. G, global. T, temporal.; TS, superotemporal. NS, superonasal. N, nasal. NI, inferonasal. TI, inferotemporal. WNL, within normal limits. BL, borderline. ONL, outside normal limits. Measured by Spectralis spectral-domain optical coherence tomography Glaucoma Module Premium Edition. Scan circle of 3.5 mm in diameter among three scan circles (3.5, 4.1, and 4.7 mm in diameter) of the Glaucoma Module Premium Edition was used for peripapillary RNFL thickness measurement.

**Table 4:** Retinal nerve fiber layer according to optic disc size.

Characteristics		Subgroups categorized by CSLO			*p* Values	
		Large Disc	Regular Disc	Small Disc	Among 3 Groups*	Post Hoc ^†^
**N (eyes)**	Region	39	45	12		
RNFL thickness (um)	G	95.08 ± 11.41	92.02 ± 9.35	91.00 ± 7.01	0.016	
	T	79.00 ± 15.09	77.38 ± 17.48	89.75 ± 14.44	**0.023**	**0.019 (2 vs. 3)**
	TS	121.97 ± 26.13	123.44 ± 26.27	134.33 ± 31.28	0.378	
	TI	134.85 ± 20.81	134.53 ± 25.12	134.75 ± 28.46	0.941	
	N	75.13 ± 15.27	69.04 ± 13.85	52.75 ± 16.71	**<0.0001**	**0.015 (2 vs. 3), <0.0001 (1 vs. 3)**
	NS	114.79 ± 30.86	110.80 ± 28.23	107.33 ± 27.88	0.5089	
	NI	100.10 ± 23.23	96.00 ± 32.66	96.50 ± 22.20	0.196	
RNFL classification (WNL/BL/ONL)	G	28/6/5	37/5/3	8/1/3	0.324	
	T	37/2/0	44/0/1	12/0/0	0.614	
	TS	24/9/6	34/6/5	11/0/1	0.133	
	TI	14/15/10	25/9/11	6/4/2	0.379	
	N	30/4/5	31/6/8	5/0/7	0.026	
	NS	34/3/2	36/8/1	9/3/0	0.613	
	NI	29/5/5	32/9/4	7/3/2	0.594	
Superior peak location (deg)		79.69 ± 16.26	79.76 ± 14.05	75.42 ± 10.73	0.552	
Inferior peak location (deg)		298.92 ± 12.67	298.62 ± 12.49	286.50 ± 29.61	0.303	
HVF category (WNL/BL/ONL)		29/5/5	33/6/6	6/4/2	0.317	
SE (D)		−2.74 ± 3.77	−2.19 ± 3.27	−4.28 ± 3.70	0.206	

CSLO, confocal scanning laser ophthalmoscopy. RNFL, retinal nerve fiber layer. G, global. T, temporal. TS, superotemporal. NS, superonasal. N, nasal. NI, inferonasal. TI, inferotemporal. WNL, within normal limits. BL, borderline. ONL, outside normal limits. HVF, Humphrey visual field. SE, spherical equivalent. D, diopter. * Among groups 1, 2, and 3: Kruskal-Wallis test. **Bold font** indicates significant *p* values (*p* < 0.05). ^†^Group 1 vs. group 2 or group 1 vs. group 3 or group 2 vs. group 3: Kruskal-Wallis test with Donn’s post-hoc test. The bold font indicates significant p values (*p* < 0.05).

**Table 5:** Retinal nerve fiber layer according to myopia.

Characteristics		Subgroups Categorized by SE			*p-*Values *	
		SE > −2.0 D	SE −2.0–−5.0 D	SE < −5.0 D	Among 3 Groups	Post Hoc ^†^
**N (eyes)**	Region	54	18	34		
RNFL thickness (um)	G	92.56 ± 9.21	89.50 ± 9.67	95.76 ±11.17	**0.028**	**0.029 (2 vs. 3)**
	T	75.59 ± 16.34	75.06 ± 7.74	89.65 ±18.98	**<0.0001**	**<0.0001 (1 vs. 3), 0.017 (2 vs. 3)**
	TS	119.44 ± 25.15	121.50 ± 23.41	132.97 ±30.16	**0.045**	**0.048 (1 vs. 3)**
	TI	126.07 ± 25.12	129.28 ± 25.30	145.68 ±16.60	**<0.0001**	**<0.0001 (1 vs. 3)**
	N	73.48 ± 14.66	68.44 ± 13.42	64.56 ±19.18	0.061	
	NS	113.15 ± 25.49	113.61 ± 30.61	110.15 ±31.72	0.969	
	NI	102.65 ± 30.31	84.00 ± 23.50	94.56 ±21.13	**0.020**	**0.017 (1 vs. 2)**
RNFL classification (WNL/BL/ONL)	G	45/6/3	9/5/4	26/3/5	**0.018**	
	T	51/2/1/	18/0/0	33/1/0	0.537	
	TS	36/10/8	12/4/2	27/4/3	0.421	
	TI	20/20/14	7/5/6	20/8/6	0.152	
	N	45/5/4	13/2/3	16/4/14	**0.001**	
	NS	48/6/0	14/3/1	27/5/2	0.327	
	NI	47/4/3	7/8/3	21/7/6	**<0.0001**	
Superior peak location (deg)		81.65 ± 14.69	81.33 ± 14.66	74.47 ±15.07		
Inferior peak location (deg)		297.43 ± 13.90	299.94 ± 11.36	2.19 ±0.85		
Disc area (mm^2^)		2.45 ± 0.58	2.40 ± 0.47	2.19 ±0.85		
HVF category (WNL/BL/ONL)		35/7/8	12/1/5	24/7/2		

SE, spherical equivalent. RNFL, retinal nerve fiber layer. G, global. T, temporal. TS, superotemporal. NS, superonasal. N, nasal. NI, inferonasal. TI, inferotemporal. HVF, Humphrey visual field. WNL, within normal limits. BL, borderline. ONL, outside normal limits. * Among groups 1, 2, and 3: Kruskal-Wallis test. **Bold font** indicates significant p values (*p* < 0.05). ^†^ Group 1 vs. group 2 or group 1 vs. group 3 or group 2 vs. group 3: Kruskal-Wallis test with Donn’s post-hoc test. Bold font indicates significant *p* values (*p* < 0.05).

**Table 6:** Retinal nerve fiber layer according to visual field test results.

Characteristics		Subgroups Categorized by HVF			*p-*Values
		WNL	BL	ONL	Among 3 Groups *
**N (eyes)**	Region	72	14	15	
RNFL thickness (um)	G	93.56 ± 10.27	92.79 ± 9.05	91.43 ± 14.98	0.350
	T	80.76 ± 17.27	81.43 ± 20.75	78.87 ± 18.89	0.414
	TS	124.94 ± 27.18	131.36 ± 24.63	122.75 ± 29.67	0.208
	TI	135.63 ± 23.95	131.36 ± 18.94	130.79 ± 28.22	0.557
	N	69.11 ± 17.00	64.36 ± 12.48	68.57 ± 17.65	0.095
	NS	112.22 ± 28.65	118.07 ± 31.50	110.11 ± 29.83	0.530
	NI	97.75 ± 29.69	94.36 ± 21.45	95.53 ± 28.70	0.924
RNFL classification (WNL/BL/ONL)	G	52/12/8	12/1/1	12/1/2	0.562
	T	70/2/0	13/0/1	15/0/0	0.506
	TS	51/12/9	13/0/1	8/5/2	0.100
	TI	29/24/19	7/5/2	9/2/4	0.481
	N	46/10/16	10/0/4	14/0/1	0.109
	NS	60/10/2	12/2/0	12/2/1	0.890
	NI	50/13/9	10/2/2	11/3/1	0.926
Superior peak location (deg)		78.78 ± 14.28	79.36 ± 15.01	77.94 ± 17.14	0.741
Inferior peak location (deg)		297.71 ± 17.00	299.86 ± 14.12	292.23 ± 42.41	0.883
Disc area (mm^2^)		2.46 ± 0.70	1.99 ± 0.48	2.31 ± 0.72	0.055
SE (D)		−2.64 ± 3.62	−3.04 ± 3.38	−2.46 ± 3.57	0.537

HVF, Humphrey visual field. WNL, within normal limits. BL, borderline. ONL, outside normal limits. RNFL, retinal nerve fiber layer. G, global. T, temporal. TS, superotemporal. NS, superonasal. N, nasal. NI, inferonasal. TI, inferotemporal. * Among groups 1, 2, and 3: Kruskal-Wallis test. **Bold font** indicates significant p values (*p* < 0.05). ^†^ Group 1 vs. group 2 or group 1 vs. group 3 or group 2 vs. group 3: Kruskal-Wallis test with Donn’s post-hoc test. The bold font indicates significant *p* values (*p* < 0.05).
